# Building Resilience: How Nurses Adapt and Thrive While Caring for Older Adults with Multimorbidity in Acute Healthcare Settings

**DOI:** 10.3390/healthcare14101408

**Published:** 2026-05-20

**Authors:** Norah M. Alyahya, Hanadi Dakhilallah, Bandar S. Alharbi, Muteb Aljuhani, Thurayya Eid, Abdulaziz M. Alodhailah, Rayhanah R. Almutairi, Waleed M. Alshehri

**Affiliations:** 1 Department of Community and Psychiatric Mental Health Nursing, College of Nursing, King Saud University, Riyadh 11451, Saudi Arabia; 2 Nursing Administration and Education Department, College of Nursing, Imam Mohammad Ibn Saud Islamic University (IMSIU), Riyadh 11564, Saudi Arabia; 3 Department of Community Health, Mental and Psychiatric Nursing, Imam Mohammad Ibn Saud Islamic University (IMSIU), Riyadh 11564, Saudi Arabia; 4 Department of Medical-Surgical Nursing, College of Nursing, King Saud University, Riyadh 11451, Saudi Arabia; 5 Community and Psychiatric Mental Health Nursing Department, College of Nursing, Princess Nourah Bint Abdulrahman University, Riyadh 11671, Saudi Arabia

**Keywords:** nursing resilience, professional adaptation, multimorbidity, older adults, acute care, professional development, interpretive phenomenological analysis, qualitative research

## Abstract

**Background**: Caring for older adults with multimorbidity represents one of the most challenging aspects of contemporary nursing practice. While research has examined clinical outcomes and care models, limited attention has been given to how nurses develop resilience and adapt their professional practice to meet these complex care demands. This study aimed to examine how nurses build resilience and adapt their professional practice when caring for older adults with multimorbidity in acute care environments. **Methods:** This qualitative study employed in-depth semi-structured interviews with 15 registered nurses across general and specialized hospital settings. Data were analyzed using interpretive phenomenological analysis to understand the essence of nurses’ adaptation and resilience-building experiences. **Results:** Three superordinate themes emerged: (1) Professional evolution through complexity navigation, (2) Emotional resilience and meaning-making in challenging care situations, and (3) Collaborative networks as sources of strength and learning. Nurses demonstrated remarkable capacity for professional growth, developing sophisticated coping mechanisms and finding meaning in their challenging work. **Conclusions:** Nurses caring for older adults with multimorbidity undergo significant professional development, building resilience through experiential learning, peer support, and meaning-making processes, though these gains are accompanied by real emotional costs, including moral distress and exhaustion. Understanding these adaptation mechanisms is crucial for supporting nursing workforce sustainability and optimizing patient care quality.

## 1. Introduction

The contemporary healthcare landscape presents nursing professionals with unprecedented challenges, particularly when managing older adults with multiple chronic conditions. Multimorbidity, affecting over 65% of adults aged 75 and older, has fundamentally transformed the complexity of nursing practice within acute care settings [1]. This demographic and epidemiological shift necessitates that nurses develop advanced competencies, adapt their clinical approaches, and cultivate resilience to navigate the physical, emotional, and cognitive demands of complex care delivery [2,3,4]. Nursing resilience, defined as the capacity to adapt and thrive despite adversity and change, has consequently gained significant traction in healthcare discourse [5]. This concept encompasses not only psychological robustness but also professional competency development and adaptive capacity in response to chronic workplace stressors [6,7,8]. For nurses specializing in geriatric acute care, resilience is a critical asset given the inherent unpredictability and heightened emotional labor associated with this patient population [9,10,11].

The recent literature emphasizes that resilience is not merely an innate individual trait but a dynamic process influenced by personal, professional, and organizational factors [12,13,14]. Personal factors include emotional regulation capacity, self-efficacy, and reflective practice habits; professional factors encompass clinical experience, specialty training, and mentorship relationships; and organizational factors involve staffing levels, leadership support, and access to debriefing opportunities. This perspective posits that resilience can be systematically developed through clinical experience, targeted education, and supportive work environments [7,9,15]. However, despite the growing prevalence of multimorbidity in acute care, limited research has examined how nurses cultivate resilience in this context. Traditionally, the nursing literature on stress and coping has focused disproportionately on negative outcomes, such as burnout, compassion fatigue, and turnover intentions [16]. While these remain critical concerns, there is an increasing recognition of the need to understand salutogenic processes, health-promoting, strengths-based, positive adaptation and growth mechanisms, that enable individuals to maintain health and well-being despite chronic stressors. In nursing, salutogenic approaches examine what helps nurses thrive in high-pressure environments rather than merely survive them [17,18].

Professional adaptation in nursing involves a continuous recalibration of knowledge, skills, and attitudes to meet evolving practice demands [19,20]. For those caring for older adults with multimorbidity, this process is exceptionally intricate, requiring the integration of clinical expertise across multiple pathologies and the mastery of nuanced communication for complex care transitions [21,22,23]. Furthermore, the social and collaborative dimensions of practice are vital to this development [24,25,26]. As nursing is inherently collective, peer support, mentorship, and interdisciplinary synergy serve as primary resources for mitigating the impact of challenging clinical scenarios [27]. Yet, a significant gap remains in understanding the intersection of these social supports with the individual’s adaptation process in the context of geriatric multimorbidity.

While extensive research exists regarding nursing stress, there is a paucity of evidence exploring the positive trajectories that allow nurses to build expertise and resilience when faced with complex patient profiles [28,29,30]. This study addresses this knowledge gap by examining the lived experiences of nurses as they navigate the demands of multimorbidity care. The findings seek to contribute to nursing science by illuminating the mechanisms of professional adaptation, offering evidence-based insights for the design of educational curricula, mentorship programs, and organizational support frameworks. Understanding these processes is particularly urgent given the current global nursing shortage and the imperative to retain a workforce capable of managing increasingly sophisticated healthcare needs. Consequently, this study aimed to explore how nurses develop resilience and adapt their professional practice when caring for older adults with multimorbidity in acute care settings, specifically by examining the processes of practice adaptation, identifying the factors contributing to resilience, exploring the role of collaborative support, and understanding how practitioners derive meaning and purpose from complex care delivery.

## 2. Materials and Methods

An interpretive phenomenological analysis (IPA) approach was employed to explore the lived experiences of nurses caring for older adults with multimorbidity. IPA is particularly well-suited for examining how individuals make sense of significant life experiences and adapt to major challenges [31].

This study is grounded in a phenomenological-interpretive paradigm, recognizing that nurses’ experiences are subjectively constructed and contextually situated. The approach acknowledges the researcher’s role in interpreting participants’ meaning-making processes while striving to remain faithful to their lived experiences.

Participants were recruited from two general and two specialized hospitals in Riyadh, Saudi Arabia, between January and June 2025. Purposive sampling captured variation in department type, facility type, and experience level. Initial contact was brokered through nurse managers; interested nurses contacted the research team directly. Of 23 nurses who expressed interest, 15 met eligibility criteria and enrolled; eight declined. No participants withdrew. Sample size was determined by information power principles analytical saturation was reached at participant 12.

Inclusion criteria included: (1) registered nurses with current acute care employment, (2) minimum six months experience caring for older adults with multimorbidity, and (3) willingness to discuss personal and professional adaptation experiences. The study excluded nurses in management positions to focus on direct care providers’ experiences.

In-depth, semi-structured interviews were conducted by N.M.A. and H.D., both of whom hold doctoral-level qualifications in qualitative research. No therapeutic or supervisory relationship existed between any researcher and participant. Participants were explicitly informed that participation or withdrawal would not affect their employment. Interview duration ranged from 35 to 65 min. The schedule was piloted with two external nurses; minor adjustments were made. Interviews were conducted in Arabic or English, with Arabic interviews professionally translated and back-translated. Interviews took place in private rooms at participants’ workplaces, were conducted individually, and were audio-recorded with written consent before verbatim transcription.

The interview approach emphasized narrative elicitation, encouraging participants to share specific stories and examples of how they had adapted their practice and built resilience over time. Follow-up questions explored the emotional, cognitive, and social aspects of these adaptation processes.

Ethical approval was obtained from the Institutional Review Board at King Saud University (Reference: KSU-HE-25-1253, approved 4 November 2025). All procedures conformed to the Declaration of Helsinki. Written informed consent was obtained from all participants. Participants were explicitly told that participation or withdrawal would have no bearing on employment or professional relationships. Interviewers provided contact details for occupational health and counseling services at the start of each interview, and a structured debrief was offered at its conclusion.

Data analysis followed Smith et al.’s [31] IPA framework, involving: (1) immersive reading and initial noting, (2) developing emergent themes, (3) searching for connections across themes, (4) moving to the next case, (5) looking for patterns across cases, and (6) Data analysis followed Smith et al.’s [31] six-stage IPA framework. Stage 1: N.M.A. read each transcript multiple times, producing exploratory notes capturing content, language, and emotional tone. Stage 2: initial notes were transformed into experiential themes using participants’ own language. Stage 3: connections were identified within each case. Stages 1–3 were repeated for all 15 transcripts (Stage 4). Stage 5: H.D. independently reviewed all transcripts; convergences, divergences, and contradictions were examined collaboratively; discrepancies were resolved through discussion with A.M.A. as arbiter. Stage 6: superordinate themes were developed by team consensus. ATLAS.ti (version 23, ATLAS.ti GmbH, Berlin, Germany) was used for data management; all interpretive decisions were made by the research team. The double hermeneutic was applied throughout. Researcher reflexivity was maintained via reflective journaling.

The analysis process emphasized both convergence and divergence in participants’ experiences, recognizing that adaptation and resilience processes might vary significantly among individuals while sharing common elements. Particular attention was paid to how participants made sense of their experiences and the meaning they attributed to their adaptation processes.

**Figure 1:**
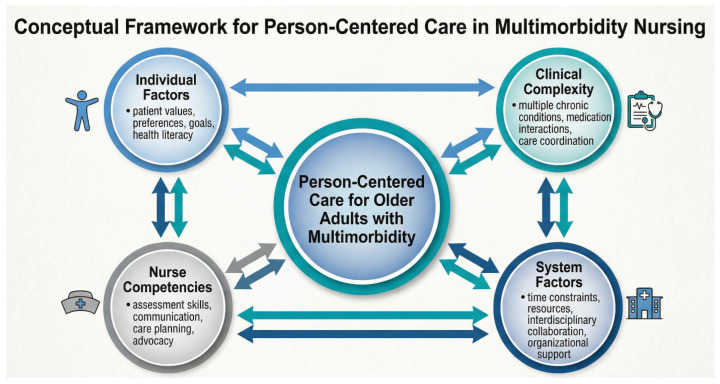
Conceptual Framework for Person-Centered Care in Multimorbidity.

This framework (Figure 1) illustrates the four interconnected domains that influence nurses’ ability to deliver person-centered care to older adults with multimorbidity in acute care environments. The bidirectional arrows represent the dynamic relationships between patient factors, nurse competencies, organizational support, and care outcomes. The framework demonstrates the complexity nurses must navigate when providing individualized care to patients with multiple chronic conditions.

Multiple strategies enhanced study credibility, including prolonged engagement with the data, peer review of analytical processes, participant validation of key findings, and reflexive examination of the researcher’s perspectives. An audit trail documented all analytical decisions and interpretive choices.

## 3. Results

### 3.1. Participant Demographic Characteristics

Fifteen nurses participated (female: *n* = 11, 73.3%; male: *n* = 4, 26.7%), ages 26–45 years. Most held a BSN (*n* = 10, 66.7%); five held an MSN (33.3%). Total nursing experience ranged from 4 to 20 years, including 2–14 years in multimorbidity care. Participants worked across Internal Medicine, ICU, and Emergency in general (*n* = 8, 53.3%) and specialized (*n* = 7, 46.7%) hospital settings. Full demographic details are in Table 1.

Analysis revealed three superordinate themes describing how nurses build resilience and adapt when caring for older adults with multimorbidity: (1) professional evolution through complexity navigation, (2) emotional resilience and meaning-making in challenging care situations, and (3) collaborative networks as sources of strength and learning. Each superordinate theme comprised two subthemes, with representative quotes illustrating participants’ experiences (see Table 2). Throughout the findings, convergent accounts and divergent or ambivalent perspectives—including accounts of moral distress, emotional exhaustion, and structural constraints—are presented to reflect the full range of experiences reported.

### 3.2. Superordinate Theme 1: Professional Evolution Through Complexity Navigation

This theme encompasses the transformative learning processes through which nurses develop expertise in multimorbidity care. Participants described their professional growth as an evolutionary process, with each challenging case contributing to their developing competency and confidence.

#### 3.2.1. Subtheme 1.1: Embracing Uncertainty as Learning Opportunity

Nurses described a fundamental shift in their relationship with clinical uncertainty, moving from anxiety-provoking situations to opportunities for learning and growth:

*“Early in my career, having a patient with five different chronic conditions would overwhelm me. Now I see each complex case as a puzzle to solve, and I’ve learned to be comfortable with not having all the answers immediately.”* (Participant 8).

This adaptation involved developing tolerance for ambiguity and building confidence through systematic assessment and problem-solving. However, less experienced nurses described ongoing discomfort:

*“I still find it hard some days. When you have a patient whose conditions are all affecting each other and you’re not sure which problem to tackle first—it’s not comfortable. I manage it better now, but I wouldn’t say I enjoy it”* (P11).

This suggests that tolerance for uncertainty develops unevenly across career stages.

#### 3.2.2. Subtheme 1.2: Developing Integrated Clinical Thinking

Participants described evolving from condition-specific thinking to integrated approaches that considered the whole person and the interactions between multiple health conditions:

*“I used to focus on treating the diabetes, then the heart failure, then the kidney disease separately. Now I think about how they all connect and affect each other, and how the person’s life circumstances influence everything.”* (Participant 4).

This evolution required developing sophisticated clinical reasoning skills and synthesizing knowledge across multiple domains while maintaining focus on individual patient needs. Two participants explicitly noted a gap in pre-registration preparation:

*“You learn about diseases one by one in nursing school. Nobody teaches you what it looks like when someone has all of them at once—and that’s what we deal with every shift”* (P6).

### 3.3. Superordinate Theme 2: Emotional Resilience and Meaning-Making in Challenging Care Situations

This theme explores how nurses develop emotional resilience and find meaning in their work with older adults with multimorbidity, often in situations involving difficult prognoses, complex family dynamics, and ethical dilemmas.

#### 3.3.1. Subtheme 2.1: Transforming Difficult Experiences into Professional Growth

Nurses described how challenging cases, rather than depleting their resources, often became sources of professional learning and personal growth:

*“There was this patient whose family was divided about his care goals, and managing that conflict was so difficult. But it taught me so much about communication and helped me develop skills I use every day now.”* (Participant 11).

This transformation was not automatic; unsupported, difficult experiences could be depleting rather than developmental.

*“Not every hard case teaches you something at the time. Some just leave you exhausted. It’s only later—sometimes months later—that you understand what you took from it”* (P12).

This suggests that organizational scaffolding, such as formal debriefing, may be necessary for growth to occur.

#### 3.3.2. Subtheme 2.2: Finding Purpose in Complexity and Suffering

Participants described developing a deep sense of purpose through their work with complex patients, often viewing themselves as advocates and sources of comfort during difficult times:

*“When I can help an elderly patient with multiple chronic conditions have a peaceful, dignified hospitalization, when I can help their family understand what’s happening—that’s when I know why I became a nurse.”* (Participant 6).

This meaning-making process appeared important for sustaining motivation and emotional well-being; however, several participants described moral distress when institutional constraints prevented them from providing the care they believed patients deserved.

*“When staffing is short and you can’t give these patients the attention they need—that is when the purpose fades. You start to question yourself”* (P3).

These accounts indicate that meaning is contingent on structural working conditions.

### 3.4. Superordinate Theme 3: Collaborative Networks as Sources of Strength and Learning

This theme describes how nurses build and utilize professional relationships as key resources for developing competency and maintaining resilience when caring for older adults with multimorbidity.

#### 3.4.1. Subtheme 3.1: Peer Learning and Knowledge Sharing

Nurses emphasized the critical role of colleague relationships in building expertise:

*“Some of my most important learning has come from other nurses who’ve been doing this longer. They teach you things you can’t learn in textbooks—like how to prioritize when everything seems urgent, or how to talk to families about difficult decisions.”* (Participant 13).

This collaborative learning involved formal mentoring relationships and informal knowledge sharing. Participants in settings without structured mentorship described relying on ad hoc peer support, which was valued but perceived as variable:

*“If you happen to work with someone experienced who is willing to help, you learn a lot. But it’s chance. Not every senior nurse has time or inclination to teach”* (P7).

#### 3.4.2. Subtheme 3.2: Interdisciplinary Collaboration as Professional Support

Participants described how working effectively with physicians, pharmacists, social workers, and other healthcare professionals not only improved patient care but also provided professional support and validation:

*“When the whole team works together on a complex case, when everyone’s expertise is valued, it makes the most challenging situations manageable. You feel supported and part of something bigger”* (P2).

Nurses in specialized hospitals more frequently described structured multidisciplinary team meetings; those in general hospitals often relied on informal communication:

*“We don’t always have a formal team meeting. You catch the doctor in the corridor, you call the pharmacist. It works, but it’s fragmented.”* (P10).

## 4. Discussion

This study provides insights into how nurses develop resilience and adapt their professional practice when caring for older adults with multimorbidity. The findings reveal adaptation as a dynamic, multidimensional process involving cognitive, emotional, and social development—one that involves real costs alongside the growth it generates. The IPA framework enabled granular attention to individual experience while identifying cross-case patterns; the person-centered care conceptual framework guided interpretation of how nurse competencies, organizational support, and care outcomes interact.

### 4.1. Professional Evolution Through Experiential Learning

The finding that nurses undergo significant professional evolution through complexity navigation aligns with Benner’s [32] novice-to-expert theory. It extends this by demonstrating how expertise develops specifically in the context of multimorbidity care. The ability to embrace uncertainty and develop integrated clinical thinking represents sophisticated professional competencies that emerge through deliberate practice and reflection.

This finding has important implications for nursing education and professional development. The observation that integrated clinical thinking develops primarily through experience is consistent with Wu et al.’s [21] scoping review, which documents the inadequacy of current healthcare education for multimorbidity care. Rather than viewing complex cases as sources of stress to be minimized, healthcare organizations should recognize them as valuable learning opportunities that contribute to professional growth when appropriate support is provided.

### 4.2. Resilience Through Meaning-Making

The transformation of difficult experiences into sources of professional growth and the ability to find purpose in complex care situations reflect what Post-Traumatic Growth theory describes as positive adaptation following challenging experiences [33,34]. For nurses in this study, the challenges of multimorbidity care became catalysts for professional development rather than sources of burnout.

Participants’ accounts of moral distress resonate with Rushton et al.’s [5] observation that resilience in high-intensity settings is contingent on alignment between professional values and structural working conditions. This finding suggests that resilience development in nursing may be enhanced through interventions that support reflection, meaning-making, and purpose identification. Organizations should implement structured debriefing processes, reflective practice initiatives, and mentorship programs and simultaneously address structural conditions, including adequate staffing, that enable meaning-making.

### 4.3. The Social Nature of Resilience

The critical role of collaborative networks highlights the fundamentally social character of nursing practice. This supports Kieft et al.’s qualitative findings that nurse work environment characteristics, including team cohesion, directly influence nurse experience and care quality. Yu et al.’s [12] updated meta-analysis similarly identified social support as one of the most consistent factors associated with nurse resilience. The observed difference between general and specialized hospitals mirrors Albarqi’s [26] findings on the benefits of systematic multidisciplinary collaboration.

The implications for practice are significant, suggesting that organizational investments in team-building, collaborative practice models, and supportive professional relationships may yield substantial returns in terms of nurse resilience and patient care quality.

### 4.4. Implications for Nursing Education and Practice

The findings carry several implications for nursing education and practice. Educational programs should incorporate complexity science principles and multimorbidity simulation scenarios. This recommendation is supported by Currie et al. [19], who demonstrated that deliberate person-centered care pedagogies shape students’ clinical reasoning.

For practicing nurses, the findings support the development of structured mentorship programs, peer support networks, and reflective practice initiatives. Organizations should foster collaborative learning, including structured multidisciplinary team meetings—as demonstrated to improve care quality in Albarqi’s [26] study—and protected peer-based case discussion time. Benadé et al. [15] similarly found that organizational support was among the strongest predictors of resilience in nurses caring for older persons.

### 4.5. Study Limitations

Several limitations should be acknowledged. The study was conducted in a specific cultural context (Riyadh, Saudi Arabia), limiting transferability to settings with different organizational structures. The purposive sample was recruited through nurse managers, which may have introduced referral bias toward nurses with more positive adaptation narratives. The cross-sectional design captures experience at a single point in time; longitudinal research would better characterize resilience trajectories. Nurses who had left practice due to burnout were not included; this survivor bias may underestimate the difficulty of sustaining practice in this context. Researcher positionality, including clinical nursing backgrounds among the team, may have shaped data collection and interpretation; these influences were mitigated through reflexive journaling and consensus analytical processes.

## 5. Conclusions

Nurses caring for older adults with multimorbidity exhibit a remarkable capacity for professional adaptation and resilience. Rather than being overwhelmed by complex care demands, many transform challenges into opportunities for growth, developing advanced competencies and deriving meaningful purpose from their work. This adaptation is inherently social, occurring through collaborative relationships and shared learning experiences.

Healthcare organizations should acknowledge the professional development potential within multimorbidity care and actively support nurses through this process. Strategies may include mentorship programs, peer learning networks, reflective practice opportunities, and the promotion of collaborative, interdisciplinary relationships.

Future research should investigate interventions that foster nurse adaptation and resilience, examine long-term career outcomes for nurses in complex care settings, and identify organizational factors that facilitate successful adaptation. Comparative studies across different healthcare systems and cultural contexts could further clarify which adaptation mechanisms are universal and which are context-specific.

Supporting nurse resilience and adaptation is crucial not only for workforce sustainability but also for patient care quality. As healthcare systems face aging populations and rising multimorbidity prevalence, investing in professional development and resilience for nurses is both a workforce imperative and a patient safety priority.

## Figures and Tables

**Table 1:** Participant demographic characteristics (N = 15).

ID	Age (Years)	Gender	Qualification	Nursing Experience (Years)	Multimorbidity Care Experience (Years)	Department	Facility Type
P1	31–35	Female	BSN	8	5	Internal Medicine	General
P2	26–30	Female	BSN	6	3	ICU	Specialized
P3	41–45	Male	MSN	15	10	Emergency	General
P4	31–35	Female	BSN	11	7	Internal Medicine	Specialized
P5	26–30	Female	BSN	5	3	ICU	General
P6	36–40	Male	MSN	14	9	Internal Medicine	General
P7	31–35	Female	BSN	9	6	Emergency	Specialized
P8	41–45	Female	MSN	20	14	ICU	General
P9	31–35	Female	BSN	7	4	Internal Medicine	Specialized
P10	36–40	Male	BSN	12	8	Emergency	General
P11	26–30	Female	BSN	4	2	ICU	Specialized
P12	36–40	Female	MSN	16	11	Internal Medicine	General
P13	31–35	Male	BSN	10	6	Emergency	Specialized
P14	31–35	Female	BSN	8	5	ICU	General
P15	41–45	Female	MSN	18	12	Internal Medicine	Specialized

BSN, Bachelor of Science in Nursing; MSN, Master of Science in Nursing; ICU, Intensive Care Unit.

**Table 2:** Superordinate Themes, Subthemes, Representative Quotes, and References.

Superordinate Theme	Subtheme	Description	Representative Quote
1. Professional Evolution Through Complexity Navigation	1.1 Embracing Uncertainty as a Learning Opportunity	Nurses shift from anxiety around uncertainty to viewing complex cases as learning opportunities, developing tolerance for ambiguity, and improving problem-solving skills.	“Early in my career, having a patient with five different chronic conditions would overwhelm me. Now I see each complex case as a puzzle to solve, and I’ve learned to be comfortable with not having all the answers immediately.” (Participant 8)
	1.2 Developing Integrated Clinical Thinking	Nurses evolve from condition-specific thinking to integrated approaches, considering interactions between multiple conditions and patient life contexts.	“I used to focus on treating the diabetes, then the heart failure, then the kidney disease separately. Now I think about how they all connect and affect each other, and how the person’s life circumstances influence everything.” (Participant 4)
2. Emotional Resilience and Meaning-Making in Challenging Care Situations	2.1 Transforming Difficult Experiences into Professional Growth	Nurses use challenging cases as opportunities for learning and professional growth through reflection and skill development.	“There was this patient whose family was divided about his care goals, and managing that conflict was so difficult. But it taught me so much about communication and helped me develop skills I use every day now.” (Participant 11)
	2.2 Finding Purpose in Complexity and Suffering	Nurses develop a sense of purpose and meaning in caring for complex patients, sustaining motivation and emotional well-being.	“When I can help an elderly patient with multiple chronic conditions have a peaceful, dignified hospitalization, when I can help their family understand what’s happening—that’s when I know why I became a nurse.” (Participant 6)
3. Collaborative Networks as Sources of Strength and Learning	3.1 Peer Learning and Knowledge Sharing	Colleagues provide critical learning opportunities and practical knowledge through both formal and informal interactions.	“Some of my most important learning has come from other nurses who’ve been doing this longer. They teach you things you can’t learn in textbooks—like how to prioritize when everything seems urgent, or how to talk to families about difficult decisions.” (Participant 13)
	3.2 Interdisciplinary Collaboration as Professional Support	Working with a multidisciplinary team enhances patient care and provides professional validation and emotional support.	“When the whole team works together on a complex case, when everyone’s expertise is valued, it makes the most challenging situations manageable. You feel supported and part of something bigger.” (Participant 2)

## Data Availability

The datasets generated and analyzed during this study are available from the corresponding author on reasonable request. The data are not publicly available due to ethical and privacy restrictions related to participant confidentiality.

## Abbreviations

The following abbreviations are used in this manuscript:

IPA	Interpretive Phenomenological Analysis
BSN	Bachelor of Science in Nursing
ICU	Intensive Care Unit
IMSIU	Imam Mohammad Ibn Saud Islamic University
KSU	King Saud University
MSN	Master of Science in Nursing
